# Diet components associated with specific bacterial taxa shape overall gut community compositions in omnivorous African viverrids

**DOI:** 10.1002/ece3.11486

**Published:** 2024-07-11

**Authors:** Malou B. Storm, Emilia M. R. Arfaoui, Phumlile Simelane, Jason Denlinger, Celine Alfredo Dias, Ana Gledis da Conceição, Ara Monadjem, Kristine Bohmann, Michael Poulsen, Kasun H. Bodawatta

**Affiliations:** ^1^ Section for Ecology and Evolution, Department of Biology University of Copenhagen Copenhagen Denmark; ^2^ Section for Molecular Ecology and Evolution Globe Institute, University of Copenhagen Copenhagen Denmark; ^3^ Department of Biological Sciences University of Eswatini Kwaluseni Eswatini; ^4^ Gorongosa National Park Goinha Mozambique; ^5^ Mammal Research Institute, Department of Zoology and Entomology University of Pretoria Hatfield, Pretoria South Africa; ^6^ Natural History Museum of Denmark University of Copenhagen Copenhagen Denmark

**Keywords:** *Civettictis*, *Genetta*, gut microbiome, metabarcoding, omnivores, southern Africa, Viverridae

## Abstract

Gut bacterial communities provide flexibility to hosts during dietary changes. Despite the increasing number of studies exploring the associations between broader dietary guilds of mammalian hosts and their gut bacteria, it is generally unclear how diversity and variability in consumed diets link to gut bacterial taxa in wild non‐primate mammals, particularly in omnivores. Here, we contribute to filling this gap by exploring consumed diets and gut bacterial community compositions with metabarcoding of faecal samples for two African mammals, *Civettictis civetta* and *Genetta* spp., from the family Viverridae. For each individual sample, we characterised bacterial communities and identified dietary taxa by sequencing vertebrate, invertebrate and plant markers. This led us to establish diet compositions that diverged from what has previously been found from visual identification methods. Specifically, while the two genera have been categorised into the same dietary guild, we detected more animal dietary items than plant items in *C. civetta*, while in *Genetta* spp., we observed the opposite. We further found that individuals with similar diets have similar gut bacterial communities within both genera. This association tended to be driven by specific links between dietary items and gut bacterial genera, rather than communities as a whole, implying diet‐driven selection for specific gut microbes in individual wild hosts. Our findings underline the importance of molecular tools for improving characterisations of omnivorous mammalian diets and highlight the opportunities for simultaneously disentangling links between diets and gut symbionts. Such insights can inform robustness and flexibility in host‐microbe symbioses to dietary change associated with seasonal and habitat changes.

## Graphical abstract

This study employs DNA metabarcoding on faecal samples to analyse the diets and gut bacterial communities of two African mammals within the order Carnivora: *Civettictis civetta* and *Genetta* spp. We demonstrate a significantly higher number of diet taxa than previous morphological studies, offering deeper insights into the feeding behaviours of these mammals. Furthermore, we show positive associations between consumed diets and gut microbiome compositions that overall tend to be driven by specific correlations between gut bacterial taxa and specific dietary items, underlining how diet acts as a driver of individual variation in wild mammalian gut microbiomes.

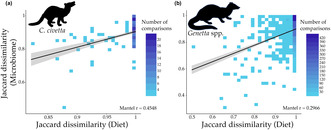

## 1 INTRODUCTION

Animals harbour a diversity of symbiotic bacteria in their digestive tracts that facilitate a variety of functions, particularly related to digestion and nutrition (Ley, Lozupone, et al., 2008; McFall‐Ngai et al., 2013). Consequently, associations between host dietary guilds and their gut bacterial community composition have been observed across the animal kingdom (Bodawatta, Koane, et al., 2021; Nishida & Ochman, 2018; Song et al., 2020). This is particularly evident in mammals, where host phylogeny, physiology and dietary guild structure gut communities (Amato et al., 2019; Ley, Hamady, et al., 2008; McCord et al., 2014; Muegge et al., 2011; Song et al., 2020; Youngblut et al., 2019), ultimately resulting in some degree of specificity in complex gut microbial communities (Mallott & Amato, 2021).

Diets of wild mammals, however, fluctuate due to seasonal variations in food item availability (De Barba et al., 2014), and most mammals do not specialise in a single diet component (Spencer et al., 2017). This implies that dietary changes are likely to lead to changes in gut bacterial communities. Studies on humans and captive mammals have demonstrated the importance of dietary macronutrients and compositions for gut microbiome structure (Amato et al., 2015; Coelho et al., 2018; Frankel et al., 2019; Ley, Hamady, et al., 2008) and the ability in microbial communities to track dietary changes of individual hosts (David et al., 2014). Similar patterns have been observed in wild mammals, where gut bacterial communities change with seasonal and environmental dietary shifts (Bjork et al., 2022; Kartzinel et al., 2019; Li et al., 2016; Ren et al., 2017) and individual variation in diet consumption (Mallott et al., 2018). However, most of this work stems from wild primates (Barelli et al., 2020; Bjork et al., 2022; Mallott et al., 2018; Sharma et al., 2020), and we lack knowledge from other wild mammalian clades, particularly in omnivores that consume a variety of dietary items.

To establish the link between variation in diet and microbiome structure in animals, characterisation of both diets and microbial communities is needed on the same focal host individuals (Baiz et al., 2023; Bodawatta et al., 2022; Mallott et al., 2018). Traditionally, diets have been primarily characterised by visual observations of feeding events or morphological identification of diet remains from gut content, faeces or regurgitated samples (Breuer, 2008; Emmons, 1987; Mallott et al., 2018). These techniques provide limited taxonomic resolution and thus tend to underestimate the dietary breadth of a species (Neilsen et al., 2017; Soininen et al., 2009; Valentini et al., 2009). DNA metabarcoding has improved this by enabling the identification of prey taxa that are not evident morphologically (Bodawatta et al., 2022; Deagle et al., 2009; Pompanon et al., 2011; Soininen et al., 2009) and reducing human‐related identification errors (De Barba et al., 2014). Results from diet metabarcoding approaches need to be evaluated with caution, as these methods can detect non‐voluntarily consumed diet items (e.g. flower mites on consumed vegetation) and/or items consumed by the prey itself (secondary consumption) (Bowser et al., 2013; Neilsen et al., 2017). Despite these caveats, the taxonomic resolution that can be obtained through metabarcoding enables deeper insights into associations between host diets and gut bacteria (Bodawatta et al., 2022). Coupling metabarcoding of diet and gut bacteria from the same individual remains absent for non‐primate wild mammals, with a few exceptions in herbivorous megafauna (Kartzinel et al., 2019; Prewer et al., 2023). This gap is particularly prominent in wild omnivorous mammals. Omnivores, with their broad dietary niches and high individual variation in dietary intake, represent optimal natural model systems to study the interactions between diet and gut microbes. Such investigations can provide novel and valuable insights into how dietary variation and diversity can impact gut bacterial communities of wild mammalian species.

**FIGURE 1:**
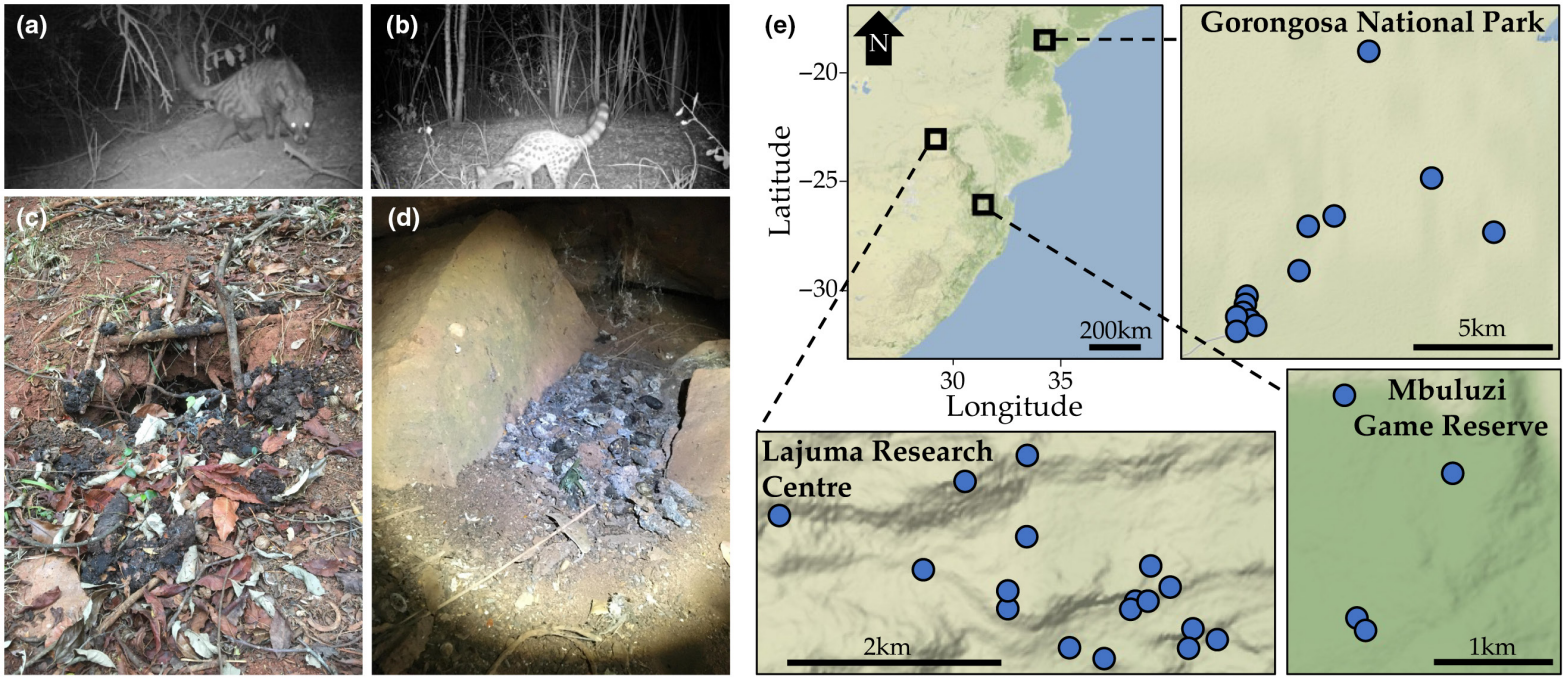
Focal genera, sample collection and maps of sampling locations. Images of (a) *Civettictis civetta*, (b) *Genetta* sp., (c) Latrine of *C. civetta*, (d) Latrine of *Genetta* sp. (e) Sampling locations in South Africa (Lajuma Research Centre), Mozambique (Gorongosa National Park) and Eswatini (Mbuluzi Game Reserve).

To help fill this knowledge gap, we investigated diet and gut bacterial composition of two closely related mammalian genera from the family Viverridae: *Civettictis* (*Civettictis civetta*, The African Civet) and *Genetta* (*Genetta* spp.) (Figure 1a–d) that belong to the order Carnivora, but feed on a diversity of plant and animal taxa. We characterised the dietary intake of individuals from three populations in Southern Africa (Figure 1e) through metabarcoding of faecal samples with primer sets targeting invertebrates, vertebrates and plants, and gut bacteria through amplicon sequencing of the v4 region of the bacterial 16S rRNA gene. We expected host genus‐specific and geographical differences in diets, as previous visual diet identification studies detected varying diets between the genera and across geographic regions (Amiard et al., 2015; Amroun et al., 2014). Further, if bacterial communities would be strongly associated with diets, we expected host genus‐ and region‐specific differences in gut microbiome composition, and that individuals with similar diets would harbour compositionally similar gut communities. Finally, if gut bacteria are associated with individual variation in diet composition, we expected links between particular dietary items and specific bacterial taxa.

## 2 MATERIALS AND METHODS

### 2.1 Study organisms

The family Viverridae (Viverrids; Order: Carnivora) consists of small, nocturnal carnivorous mammals (Carvalho et al., 2016). Of these, those that inhabit southern Africa include the African civet (*Civettictis civetta*) and several species in the genus *Genetta* (e.g. *G. maculata* and *G. genetta*) (Figure 1) (Carvalho et al., 2016; Roux et al., 2016; Swanepoel et al., 2016; Veron, 2007; Widdows et al., 2016). Previous research found that *Genetta* spp. feed mainly on rodents (Roberts et al., 2007; Rosalino & Santos‐Reis, 2002), while *C. civetta*, despite having a varied diet, feeds mostly on various fruits (Amiard et al., 2015; Mullu & Balakrishnan, 2015). These species use latrines as defecation sites and return to these regularly (Abiyu et al., 2015; Mullu & Balakrishnan, 2015; Sánchez et al., 2008), making it possible to monitor activity in the latrine, while ensuring collection of fresh faeces (Figure 1a–d).

### 2.2 Study sites and sample collection

Faecal samples were collected at three locations from *C. civetta* and *Genetta* spp. in southern Africa (Figure 1d). Samples were collected at Lajuma, a part of the Soutpansberg Mountain range within the UNESCO Vhembe Biosphere Reserve (23°02′ S, 29°26′ E), South Africa (Willems & Hill, 2009), Mbuluzi Game Reserve (26°05′‐26°22′ S 31°45′‐32°10′ E) in the Mlawula‐Mbuluzi reserve network in the north‐eastern Eswatini (Monadjem & Reside, 2008), and Gorongosa National Park in central Mozambique (18°45′58″ S 34°30′00″ E), comprising 4000 km^2^ of protected area (Correia et al., 2017; Rodrigues et al., 2017). Three additional samples were collected by collaborators in Medike Mountain Reserve and Blouberg Nature Reserve, South Africa, close to Lajuma. All of these study areas have only one *Civettictis* species (*C. civetta*), but there are at least two known species of *Genetta*. Our sample sites in Eswatini and Mozambique are inhabited by *G. maculate*, while sample sites in South Africa are inhabited by both *G. maculata* and *G. genetta* (Kingdon & Hoffmann, 2013).

Latrines were located and monitored every 2–3 days, from the start of September to mid‐December 2017, resulting in 119 fresh faecal pellets: 77 in South Africa, 22 in Mozambique and 20 in Eswatini (Table S1). To avoid environmental contamination, no sample was collected after rainfall and faecal material was collected from the core of the pellets (avoiding the material that has been in contact with the environment) (King et al., 2008). Ethanol and flame‐sterilised forceps were used for sampling, and latex gloves were used during collection. Each sample was divided into three sub‐samples that were placed in separate 2 mL Eppendorf tubes containing RNAlater®, composed of 25 mM Sodium Citrate, 10 mM EDTA, 70 g ammonium sulphate/100 mL solution (pH 5.2) and stored at 5°C (Table S2). To create DNA reference sequences for host species determination, approximately 1 × 0.5 cm skin samples were acquired from the skin collection at the Natural History Museum of Denmark, from specimens of *C. civetta*, *Genetta tigrina* and *Genetta genetta* and the Mongoose species *Cynictis penicillata*, *Helogale parvula*, *Bdeogale crassicauda*, *Galerella sanguinea* and *Mungos mungo* (Table S3).

### 2.3 DNA extractions

The QIAamp® PowerFecal® DNA kit (Hilden, Germany) was used for DNA extraction from the 119 faecal samples and the Qiagen DNeasy® blood and tissue kit (Hilden, Germany) was used for extractions from skin samples. Both protocols were used following the manufacturer's guidelines with the following alterations, applied to both kits. Proteinase K (5 μL per reaction) was added with the lysis buffer, and lysis time was increased to appx. 22 h. The Elution buffer was replaced with a Tris‐EDTA buffer with TWEEN®20 (TET‐buffer). Fifty microlitres of TET buffer were added to the Spin Column, followed by 15 min incubation at 37°C. This step was repeated one time before centrifuging, resulting in a total elution volume of 100 μL. One negative extraction control was included per five biological samples to screen for contamination.

### 2.4 Metabarcoding for diet analysis

We used SYBR Green ROX quantitative PCRs (qPCR) to screen for inhibition within our DNA extracts and to identify optimal number of PCR cycles required for each of the five dietary primer sets (Table 1), following Murray et al. (2015) and Schnell et al. (2015). Specifically, qPCRs were conducted on a subset of sample DNA extracts and the negative extraction controls using the following primers targeting vertebrates; 16S mamF and 16S mamR (90 bp excluding primers) (Taylor, 1996) and RiazF and RiazR (97 bp excluding primers) (Riaz et al., 2011), invertebrates; Coleop_16Sc and Coleop_16Sd (105 bp excluding primers) (Epp et al., 2012) and ZBJ‐ArtF1c and ZBJ‐ArtR2c (157 bp excluding primers) (Zeale et al., 2011) and plants; Trac01 and ITS 7A (267 bp excluding primers) (Taberlet et al., 2018) (Table 1). We utilised these five primers to capture as much as dietary taxa diversity, while accounting for potential primer biases (c.f., Alberdi et al., 2017). To screen for inhibition, qPCRs were run in a dilution series – neat, 1:5 and 1:10 dilutions. The 25‐μL reactions included 1‐μL DNA template and 1x AmpliTaq Gold™ PCR buffer (Applied Biosystems, Waltham, MA, USA), 2.5 mM MgCl_2_, 0.2 mM dNTPs, 1 unit Taq Gold™ (Applied Biosystems, Waltham, MA, USA), 0.5 mg/mL BSA, 0.6 μM forward primer, 0.6 μM reverse primer and 1 μL SYBR green mix (1:4 SYBR green (S7563) (Invitrogen) to ROX reference dye (12223–012) (Invitrogen, with 200 parts high grade DMSO) per reaction). qPCRs were carried out with conditions specified in Table 1, followed by a melting curve and no extension step. Based on the qPCR screening, the use of 1:10 diluted template and 40 cycles was chosen for the following metabarcoding PCR amplifications. For these, the reagent mix was the same as for the qPCR, although excluding the SYBR Green ROX. Extraction and PCR negative controls were included. PCRs were carried out using the tagged PCR approach, where primers carry 5′ nucleotide tags, which allow for pooling after a single PCR step (Binladen et al., 2007; Bohmann et al., 2022). We used unmatching tags, meaning that forward and reverse tags did not match (e.g. F1‐R2, F2‐R3). For each sample, negative and positive controls (16S mam: *Canis lupus*, Riaz: *Canis lupus*, Coleop_16S: *Tenebrio molitor*, ZBJ‐Art: *Tenebrio molitor*, Trac01: *Galanthus* spp.) and three PCR replicates with unique tag combinations were produced. No nucleotide primer‐tag combination was repeated in replicates of the same samples or in the same amplicon pool. All faecal samples were amplified using all five primer sets with their respective PCR parameters (Table 1), whereas skin samples were only amplified using the 16S mam primer set. PCR products were visualised on 2% agarose gels. Samples with no bands were reamplified and discarded if no band appeared after several trials. Amplified samples were pooled approximately equimolar, according to band intensity (5 μL for bright, 10 μL for medium and 15 μL for weak). Fifteen microlitres of each negative control with no band were added to the library pools. However, if a band were present, they were pooled according to the band strength as was done for samples. PCR products were pooled into 10 pools, two pools per primer set (no identical tag‐combinations were present in the same pool). Amplicon pools were bead‐purified using SPRI beads (Rohland & Reich, 2012) and libraries were built using the PCR‐free Tagsteady library protocol (Caroe & Bohmann, 2020) and quantified using NEBNext® Library Quant Kit for Illumina® (New England BioLabs). Libraries were sequenced with 250 bp paired‐end read length and 7–8% PhiX on an Illumina MiSeq platform aiming for 25,000 reads per PCR replicate.

**TABLE 1:** Primers and overview of consisting taxa, target gene, marker length, forward and reverse sequences, and suitable qPCR and PCR parameters for each primer.

Primer name	Target taxa	Target marker	Forward primer sequence	Reverse primer sequence	qPCR/PCR temperature profile	No. of cycles	References
F: 16S mamF R: 16S mamR	Mammalia	16S rDNA	CGGTTGGGGTGACCTCGGA	GCTGTTATCCCTAGGGTAACT	95°C – 10 min 95°C – 12 s 59°C – 30 s 70°C – 25 s 72°C – 7 min	40	(Taylor, 1996)
F: Trac01 R: ITS‐7A	Tracheophyta	ITS1	GATATCCRTTGCCGAGAGTC	GAAGGAGAAGTCGTAACAAGG	95°C – 10 min 95°C – 30 s 56°C – 30 s 72°C – 30 s 72°C – 7 min	40	(Taberlet et al., 2018)
F: RiazF R: RiazR	Vertebrata	12S rDNA	TTAGATACCCCACTATGC	TAGAACAGGCTCCTCTAG	95°C – 10 min 94°C – 30 s 51°C – 30 s 72°C – 60 s 72°C – 5 min	40	(Riaz et al., 2011)
F: ZBJ‐ArtF1c R: ZBJ‐ArtR2c	Arthropoda	COI	AGATATTGGAACWTTATATTTTATTTTTGG	WACTAATCAATTWCCAAATCCTCC	95°C – 10 min 95°C – 15 s 52°C – 30 s 72°C – 30 s 72°C – 7 min	40	(Zeale et al., 2011)
F: Coleop_16Sc R: Coleop_16Sd	Coleoptera	16S rDNA	TGCAAAGGTAGCATAATMATTAG	TCCATAGGGTCTTCTCGTC	95°C – 10 min 95°C – 15 s 55°C – 30 s 72°C – 30 s 72°C‐ 7 min	40	(Epp et al., 2012)
V4.SA504 V4.SB711	Bacteria	V4 region of the 16S rRNA	AATGATACGGCGACCACCGAGATCTACACCTGCGTGTTATGGTAATTGTGTGCCAGCMGCCGCGGTAA	CAAGCAGAAGACGGCATACGAGATTCAGCGTTAGTCAGTCAGCCGGACTACHVGGGTWTCTAAT	94°C – 4 min 94°C 30 s 56°C – 30 s 72°C – 30 s 72°C – 4 min	35	(Otani et al., 2016)

### 2.5 16S rRNA amplicon sequencing for gut microbiome characterisation

To characterise gut bacterial composition, DNA extracts were first screened for bacterial DNA by conducting PCR amplifications with the primers V4.SA504 and V4.SB711 that target the V4 region of the 16S rRNA gene (250 bp) (Kozich et al., 2013). These PCRs were performed in 20‐μL reactions, including 1 μL of template DNA, 8.5 μL of REDTaq ReadyMix (Sigma‐Aldrich, USA), 8.5 μL of sterile distilled water, 1 μL each from 10 μM forward and reverse primers. PCRs were carried out using conditions specified in Table 1 (Otani et al., 2016). Amplification success was checked on a 1.5% agarose gel. Of the 119 sample extracts, 112 amplified successfully and their DNA extracts were sent for MiSeq amplicon sequencing at an Illumina platform (250 bp paired‐end) at the Microbiome Core at the University of Michigan, USA, prepared using the approach described in (Kozich et al., 2013).

### 2.6 Bioinformatics and data analysis

Analyses of diet data (i.e. adaptor and quality trim and merging of paired‐end reads) were carried out using AdaptorRemoval version 2 (Schubert et al., 2016). A modified version of the DAMe pipeline (Yang et al., 2021) was used to sort sequences. Sequences were sorted according to primer sequence and assigned tag combinations from PCRs. After sorting, sequences were filtered and only sequences that were present in minimum two out of three PCR replicates per sample were kept. This strict approach was employed to ensure that the identified sequences were true detections and not caused by, for example PCR biases. Further, the minimum sequencing length was set at 80 bp for 16Smam F and R, 250 bp for Trac01 and ITS 7A, 80 bp for Riaz F and R, 140 bp for ZBJ‐ArtF1c and ZBJ‐ArtR2c and 90 bp for Coleop_16Sc and Coleop_16Sd. Sequences were clustered using Sumaclust with a similarity cut‐off of 0.97, and a contingency table of operational taxonomic units (OTUs) was created for each of the five primer sets. Then the reads of each sample were normalised by scaling to 50,000, creating five different OTU tables. Taxonomy was assigned to OTUs (Altschul et al., 1997) by blasting them to the NCBI GenBank database using blastN (accessed April 2020), with a 97% similarity match. The LULU algorithm was used to remove erroneous OTUs (Froslev et al., 2017). We blasted the sequences against each other with percent query coverage per hsp set to 80 and percent identity set to 84. Hereafter, LULU was run with the following settings: minimum_ratio_type=“min”, minimum_ratio=1, minimum_match=84, minimum_relative_cooccurence=0.95. The output was imported into Megan version 6.12.3, with standard LCA parameter settings, except for minimum percentage identity, which was set to 90% (Huson et al., 2007). OTUs assigned to humans, fungi, bacteria, nematodes and mites were removed from the data sets. Any OTUs present in any of the negative controls were removed from the dataset. These removed OTUs included genus *Macrotermes* (one blank) from Coleop_16S primers; order Sarcoptiformes (one blank) from ZBJ‐Art primers; *Homo sapiens* (six blanks), order Carnivora (one blank) and family Felidae (one blank) from 16Smam primers; *Homo sapiens* (nine blanks), and family Felidae (one blank) from Riaz primers; genus *Morus* (two blanks), *Thyrsodium puberulum* (two blanks), and genus *Solanum* (one blank) from Trac01 primers. Twenty‐eight samples with host identity that were unknown or not assigned to *C. civetta* or the genus *Genetta* were omitted from the analyses. Furthermore, one sample was removed due to an error in the original 16S rRNA bacterial sequencing file and 10 were removed as no dietary OTUs were classified, leaving 80 samples for downstream analysis. After the taxonomic classification, we merged the five OTU tables to acquire one dietary taxa table, which was used for subsequent analyses.

Bacterial 16S rRNA sequences were processed using the DADA2 pipeline (Callahan et al., 2016) in RStudio v.4.0.3 (Team, 2022). The reads were filtered, using the filterAndTrim command with default parameters (truncLen = c(225,160), maxN = 0, truncQ = 2, rm.phix = TRUE, maxEE = 2). After merging the paired reads, amplicon sequence variants (ASVs) at 100% similarity were generated and chimaeras were removed using the removeBimeraDenovo command. ASVs were assigned to taxonomy using the Silva v.138.1 reference database (Quast et al., 2013; accessed on January 4th, 2022). We removed archaeal, mitochondrial and chloroplast sequences from the ASV table.

Species accumulation curves (Figure S1) were generated to assess if the sample depth was sufficient to cover expected diversity (Wynne et al., 2018), using the vegan package (Oksanen et al., 2022). Frequency of occurrence was calculated to investigate the frequency of different dietary items among individuals. All analyses of diets were conducted on presence/absence of taxa, as the use of relative abundances can be misleading due to risks of biological and laboratory factors that skew the results (e.g., biomass and tissue content differences of dietary items (c.f., Alberdi et al., 2019), primer biases (c.f., Alberdi et al., 2017)) and the merging of OTU tables from different primer sets. Analyses based on taxonomic identification were limited to order level, as the majority of taxa were only classified to order or higher taxonomy. Data from *C. civetta* and *Genetta* spp. were treated separately, except for analyses examining overall dietary taxa and microbial diversities and compositions. All statistical analyses and visualisations were done in RStudio v.4.0.3 (Team, 2022). Alluvial plots to visualise diet proportions were generated using the ggalluvial (Brunson & Read, 2023) and ggplot2 (Wickham, 2016) packages.

To investigate the influence of host species and sample location and their interaction on gut microbiomes and consumed diets we conducted Permutational Multivariate Analysis of Variance (PERMANOVA) using the adonis2 function with ‘by’ parameter set to ‘margin’ in the vegan package (Oksanen et al., 2022). We used Bray–Curtis dissimilarity distance matrix for the gut bacterial communities and Jaccard (unweighted) dissimilarity distance matrix for diet with 999 permutations. The same distance matrixes were used for computing Principal Coordinate Analysis (PCoA) using the phyloseq (McMurdie & Holmes, 2013) and ggplot2 (Wickham, 2016) packages and the outcome of PCoAs were visualised using the scatterplot3d package (Ligges & Mächler, 2003). The vegan package was used to compute species richness for both gut microbiome and diet and was tested for normality using a Shapiro–Wilk test of normality and tested for homogeneity using a Bartlett test of homogeneity of variance. As the data was normally distributed but not homogeneous, a Welch one‐way test was used to test for difference in species richness between different categories.

To test whether diet richness was correlated with gut bacteria richness, we used the cor_test (method=”spearman”, exact = FALSE) function from R standard statistics. To investigate the association between the overall bacterial community and diet similarity, we conducted mantel tests using Jaccard dissimilarity distance matrices with 10,000 permutations in the vegan v2.5.7 package (Oksanen et al., 2022). A heatmap of the Jaccard dissimilarity with a trendline was plotted using ggplot2 v3.3.5 (Wickham, 2016). Finally, to explore whether different consumed diet items were associated with specific bacterial taxa, we conducted Pearson's correlations between relative abundance of bacterial taxa at the genus level and the proportion of different dietary taxa at the order level in individual diets using the cal_cor function in the microeco package (Liu et al., 2021). These analyses were done individually for the plant, vertebrate and invertebrate results and significance of correlations were adjusted using the false discovery rates. Correlations between diet orders and bacterial genera were visualised using Cytoscape 3.10.2 (Shannon et al., 2003).

## 3 RESULTS

### 3.1 Host identification

The 16S rRNA marker did not show any sequence variation between skin samples from *Genetta genetta* and *Genetta tigrina* (Table S3). This meant that we could not distinguish between *Genetta* species, and we therefore hereafter refer to all samples from the genus as being from *Genetta* spp.

### 3.2 Diet composition differed between species and among geographic locations

We obtained diet information from 62 samples from *Genetta* spp. and 18 from *C. civetta*, which includes 45 *Genetta* spp. and nine *C. civetta* samples from South Africa, three *Genetta* spp. and nine *C. civetta* from Mozambique, and 14 *Genetta* spp. samples from Eswatini (Table S1). The diets of both genera included vertebrates, invertebrates and plants. For *Genetta* spp., we identified 253 taxonomically assigned taxa (diet components), spanning 44 taxonomic orders, 65 families, 88 genera and 48 species. We identified far fewer taxa from *C. civetta* samples, with only 105 taxa from 32 orders, 40 families, 30 genera and 11 species (Table S4; For individual OTU tables for the 5 primer pairs, see Tables S5–S9). However, this overall reduced number of dietary taxa can be a result of the lower number of *C. civetta* samples. All diet orders that were detected were known to the areas from where samples were collected. Species accumulation curves indicated that more samples would likely provide a better representation of diet compositions (Figure S1).

**FIGURE 2:**
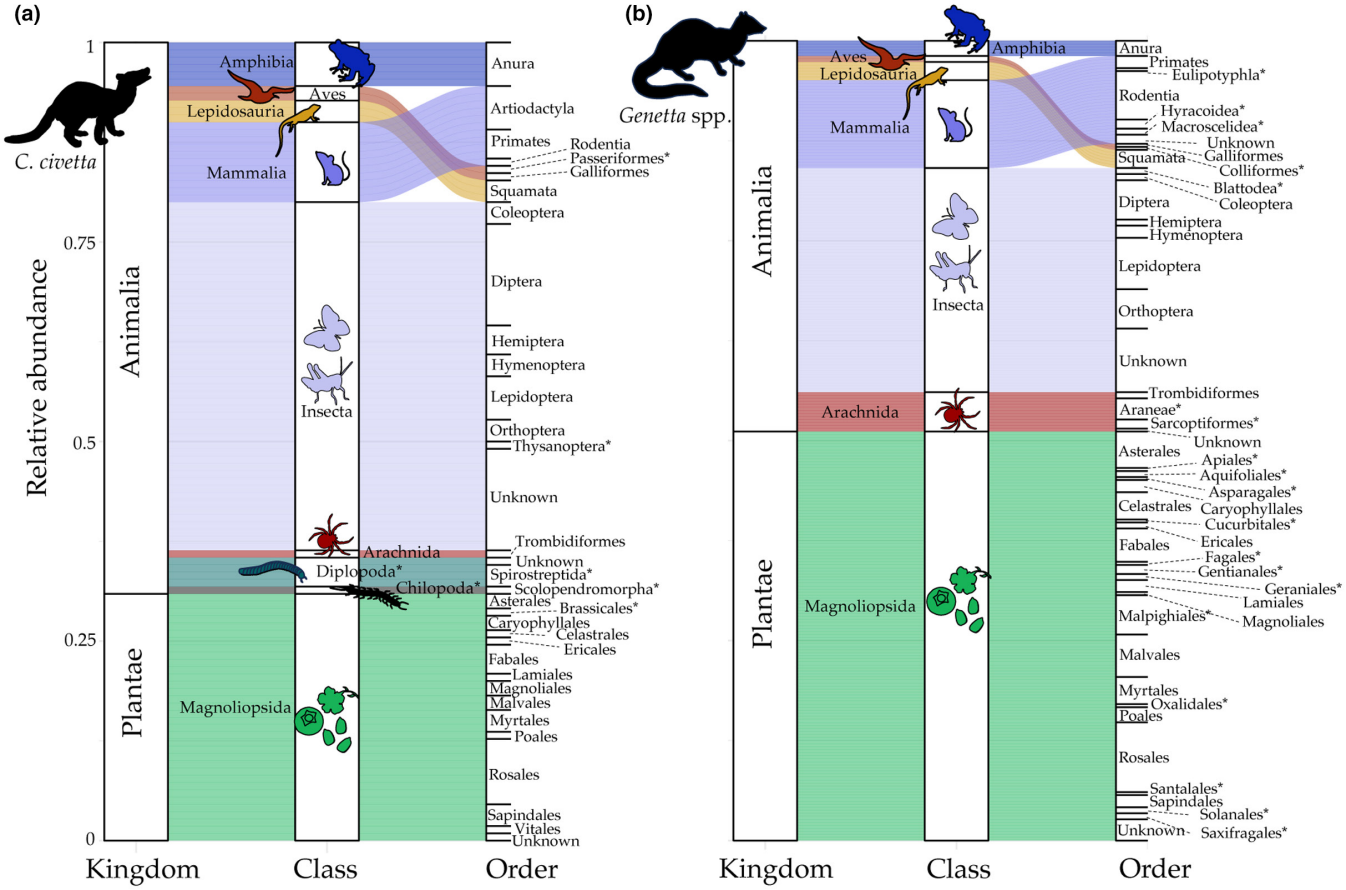
Proportions of dietary taxa (to the order level) detected in *C. civetta* and *Genetta* spp. Alluvial plots visualising the relative frequency of taxa in the diet of (a) *C. civetta* and (b) *Genetta* spp. Dietary orders only found in one host genus are indicated with asterisks. Taxa that did not assign to an order from each taxonomic class are given as ‘unknown’.

The diet of *Genetta* spp. contained more plant taxa (53.0% of taxa) (Figure 2a), while *C. civetta* diet contained more invertebrates and vertebrates (59.0% of taxa) (Figure 2b). As measured by the number of different taxa, members of the order Rosales were most frequent among the plants consumed by *Genetta* spp., but their diets also included insects (predominantly Lepidoptera) and mammals (predominantly Rodentia) (Figure 2a). *C. civetta* consumed more insect taxa (predominantly Diptera) followed by plants (predominantly Rosales) and mammals (predominantly primates, probably carcasses (Wadley, 2020)). For both *Genetta* spp. and *C. civetta*, the most frequent diet component was the genus *Ficus* within the Rosales, which was present in 38.0% of *Genetta* spp. and 19.0% of *C. civetta* samples (Table S4).

For both host genera, we observed differences in consumed diets between the sampling regions (two locations for *C. civetta* and three locations for *Genetta* spp.). For *Genetta* spp. in South Africa, a large portion of the dietary taxa was represented by multiple plant orders and rodents (Table S4). Similarly, the diet of *Genetta* spp. from Eswatini had more plant taxa, which were less frequent in Mozambique (Table S4). The diet of *C. civetta* contained both vertebrates and invertebrates, the latter most frequently being insects in both South Africa and Mozambique (Table S4). *C. civetta* diets differed in plant orders by location, with the highest number (11 orders) in samples from Mozambique (Table S4).

**FIGURE 3:**
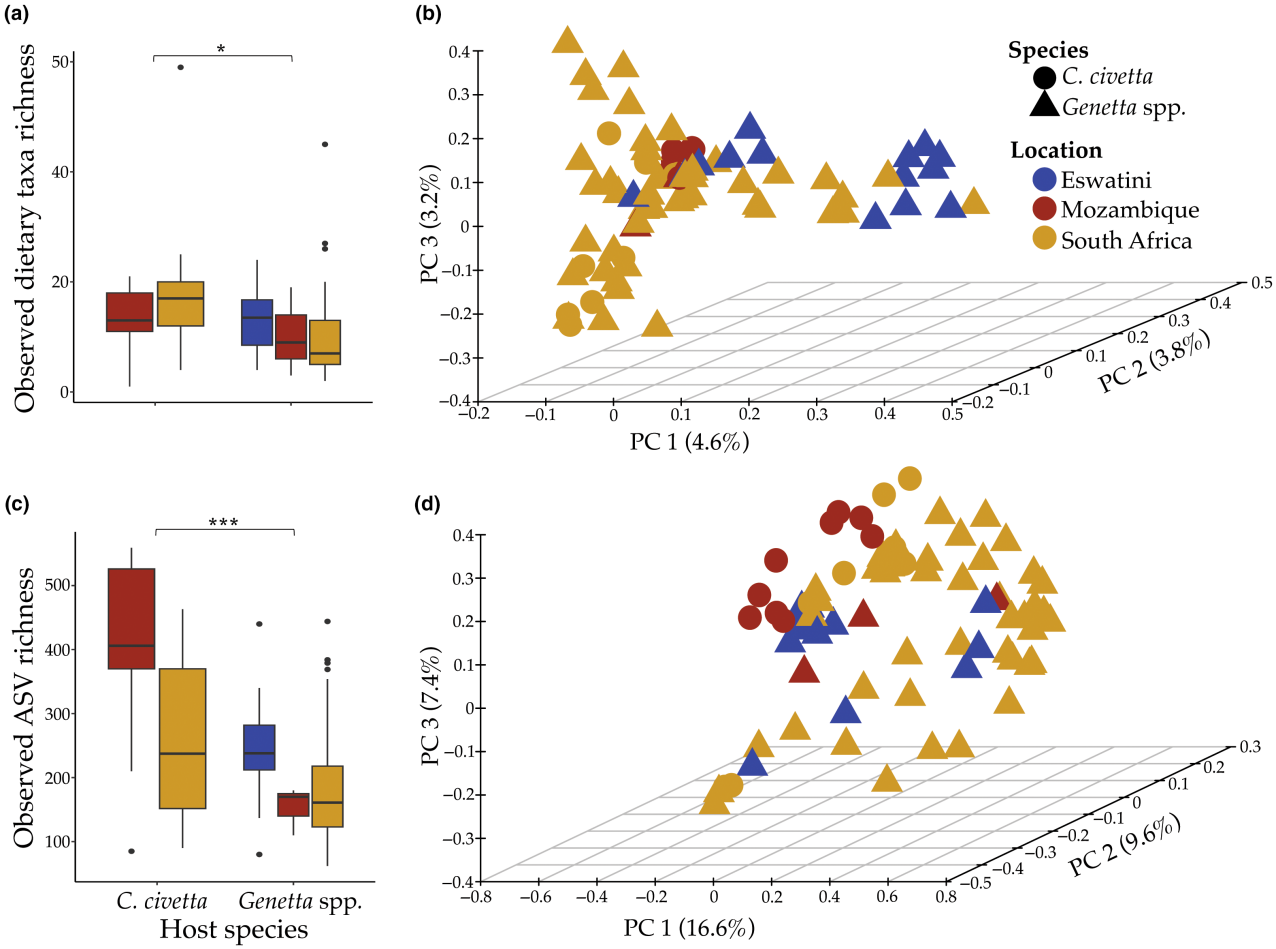
Diet and gut bacterial diversity and composition in *C. civetta* and *Genetta* spp. Box plots demonstrate the diet taxa richness (a) and ASV richness (c) in the two host species. Colours indicate the sampling localities and * indicating significance level *p* < .05 and ***p < .001. 3D PCoA ordination plots demonstrate the composition of (b) diet (based on Jaccard dissimilarity distance) and (d) gut microbiomes (based on Bray–Curtis dissimilarity distances) for *C. civetta* (circles) and *Genetta* spp. (triangles) at the three collection locations.

Overall, diet item richness was significantly higher in *C. civetta* than *Genetta* spp. (ANOVA: *F*_1_ = 5.978, *p* = .0167); however, it varied by sampling locations (Figure 3a). We observed a significant interaction between host genus and sampling location on diet composition (PERMANOVA_Host × Location_: *F* = 1.288, *R* ^2^ = 0.0157, *p* = .0084), indicating that the influence of host species on diet composition varies geographically. This aligns with the taxa‐level dietary differences observed for both genera across sampling locations (Table S4). The relative contribution of host genus and sampling locations on diet composition disappeared when their interaction was present in the model.

### 3.3 Diverse gut bacterial communities of viverrids differed between host species and locations

We obtained 17 successfully sequenced gut bacterial community samples from *C. civetta* and 60 from *Genetta* spp. From *C. civetta* we acquired 1,510,564 bacterial sequences (average ± SD: 88,856 ± 10,812) that belonged to 3508 ASVs (Table S10). *Genetta* spp. samples contained 4,509,590 sequences (average ± SD: 75,159 ± 15,531) belonging to 5141 ASVs. Overall, we identified 38 bacterial phyla, dominated by Firmicutes (45.0%), followed by Proteobacteria (23.6%), Bacteroidetes (12.6%) and Fusobacteria (11.9%). Less than 0.1% of the sequences could not be classified to a phylum (Table S11). Species accumulation curves indicated that the number of sequences per sample was insufficient to fully capture gut bacteria diversity (Figure S1).

ASV richness was significantly higher in *C. civetta* than *Genetta* spp. (Welch one‐way test: *F*_1_ = 58.61, *p* < .0001; Figure 3c). We observed a significant interaction between host genera and sampling location (PERMANOVA_Host x Location_, *F*_4_ = 1.603, *R* ^2^ = 0.0195, *p* = .0101), indicating that the structure of microbiomes differed between genera depending on their sampling location (Figure 3d). Similar to the diet composition, the interaction between host genus and sampling location removed the relative effects of individual factors.

### 3.4 Diet items and gut bacterial taxa were strongly associated with one another

**FIGURE 4:**
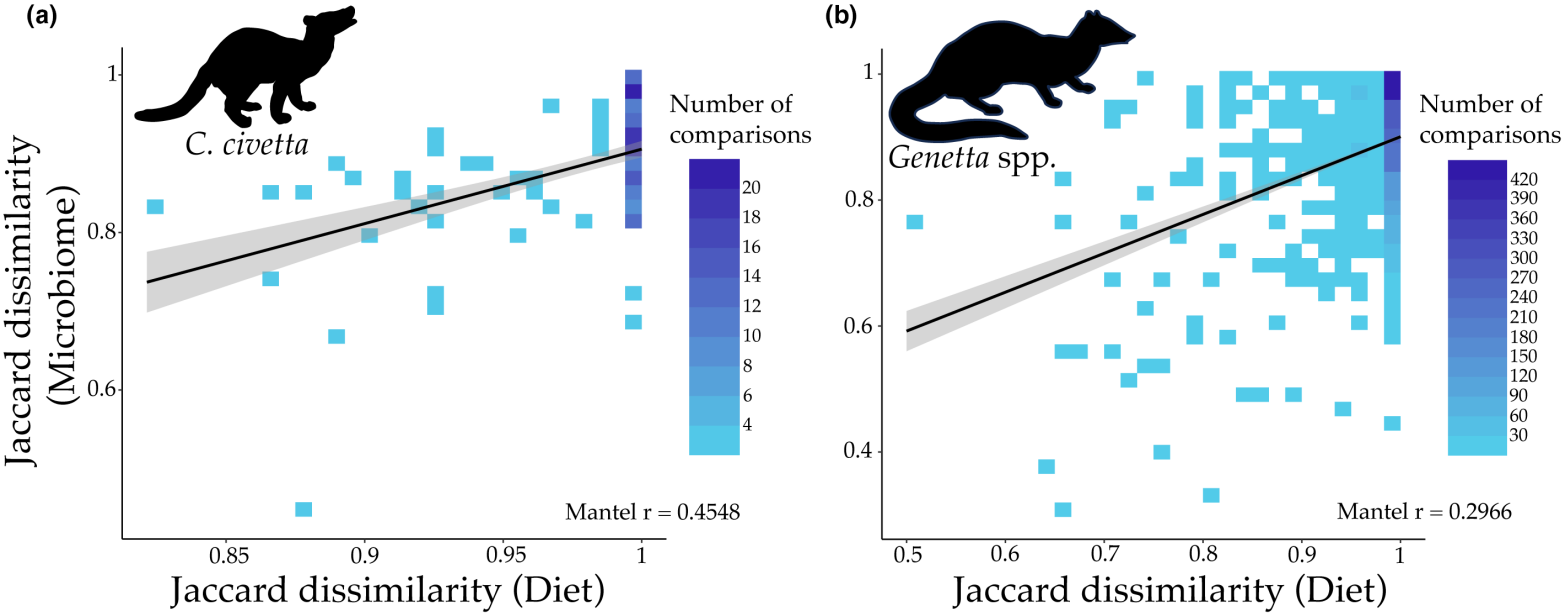
Association between diet composition with microbiome composition. Relationship between the gut bacterial and consumed diet dissimilarity (measured with Jaccard dissimilarity distances) in (a) *C. civetta* and (b) *Genetta* spp. Mantel correlations for each genus are given within the panels. The grey area around each trendline is the 95% confidence interval (note that this trendline is only for visualisation and that the statistics were conducted using Mantel tests). Coloured squares in the heatmap show the comparison of two samples with their dissimilarity value for both the diet (x‐axis) and the gut bacteria (y‐axis). The legends show the number of comparisons between samples for a given square in the plot.

For 17 *C. civetta* individuals and 60 *Genetta* spp. Individuals, we had both diet and microbiome data. In *C. civetta*, we did not find a correlation between diet item richness and ASV richness (Pearson's correlation, *ρ* = 0.0450, *p* = .864). In contrast, we observed a significant correlation in *Genetta* spp. (*ρ* = 0.3100, *p* = .0145). However, this correlation tended to be sampling location‐specific as we only detected a significant correlation in Eswatini (*ρ* = 0.8500, *p* = .0001) and not in South Africa (*ρ* = 0.0950, *p* = .5450). Mantel tests indicated significant positive correlations between diet similarity and gut bacterial community similarity in both *C. civetta* (Mantel *r* = 0.4548, *p* = .0002) and *Genetta* spp. (Mantel *r* = 0.296, *p* < .0001; Figure 4). *C. civetta* sample SA197 was an outlier with an extremely low number of diet item and gut microbes shared with other individuals, so we removed it from this analysis. Overall, our findings suggest that the composition of consumed diets is associated with the composition of gut bacterial communities in both host genera.

**FIGURE 5:**
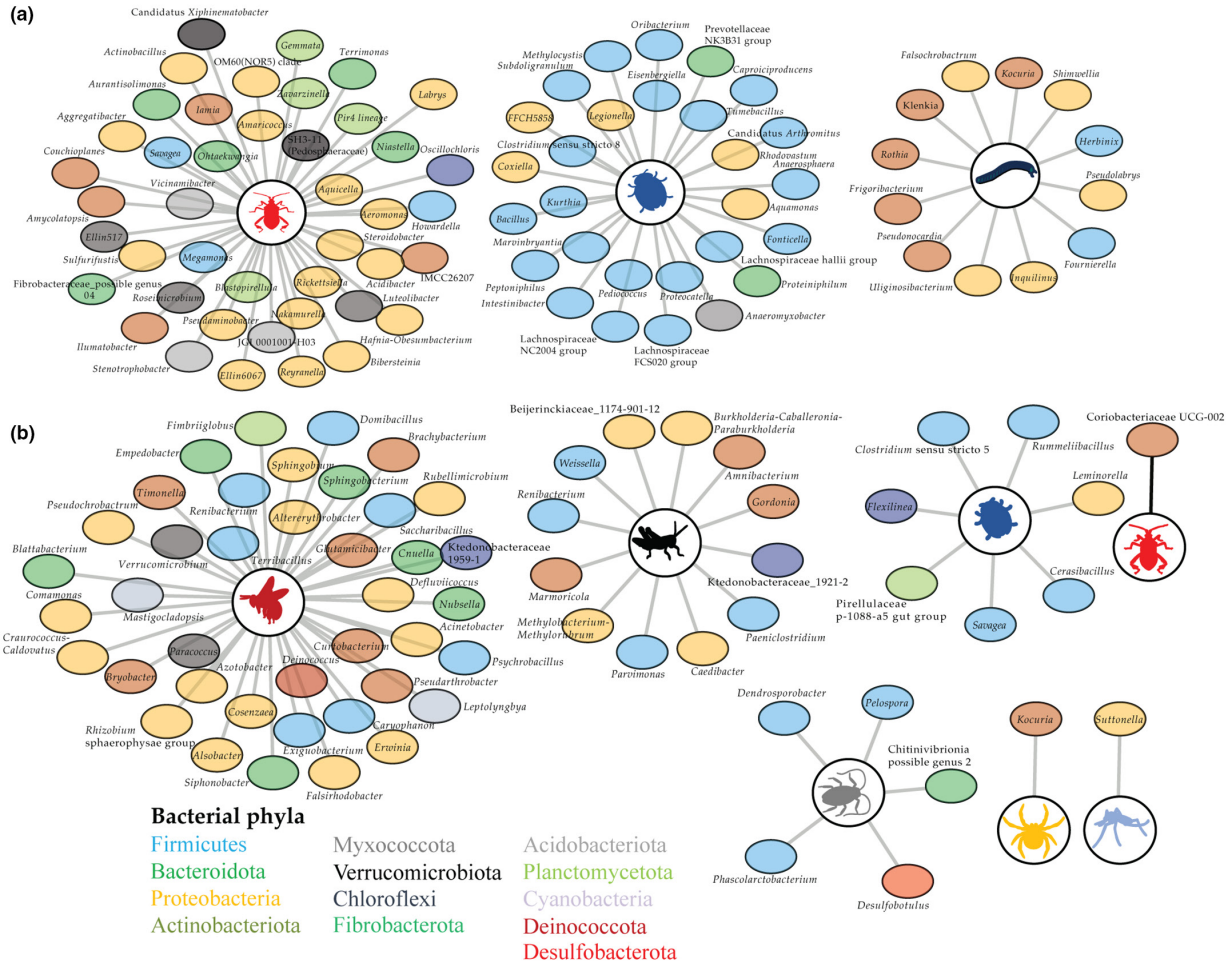
Associations between proportions of invertebrate dietary items (order level) and relative abundance of bacterial taxa (genus level) in (a) *C. civetta* and (b) *Genetta* spp. Figure only depicts Pearson's correlations with *p* < .0001 (***). Full list and correlations between vertebrate and plant dietary items and bacterial genera in Table S12. Networks were generated using the output files from the correlations.

The overall associations between diet and bacterial community similarities tended to be driven by relations between specific bacterial taxa and the proportion of dietary items in individual diets. We observed specific sets of bacterial genera to be associated with proportions of specific order‐level dietary items in both *C. civetta* and *Genetta* spp. However, the taxonomic identity of bacterial taxa associated with taxonomically‐similar diet items differed between the two host genera. All significant Pearson's correlations were positive. In *C. civetta*, there were 144 significant correlations between different bacterial genera and invertebrate orders (Figure 5, Table S12), for which most were with Hemiptera (57) and Coleoptera (38). In *Genetta* spp., there were 91 significant correlations of which Hymenoptera accounted for the majority (47) (Figure 5, Table S12). We found 276 significant correlations between vertebrate orders and bacterial genera in *C. civetta*, dominated by associations with Artiodactyla (113), Rodentia (47) and Galliformes (60) (Table S12). In *Genetta* spp., the 172 significant correlations were most frequently with Coliiformes (48) and Macroscelidea (41). Lastly, there were 408 and 339 significant correlations between plant orders and bacterial genera in *C. civetta* and *Genetta* spp., respectively. These interactions imply that diet variation is a driver of individual variation in gut communities in wild viverrids.

## 4 DISCUSSION

Through characterising both consumed diets and faecal bacterial communities, we investigated associations between diet and gut microbiomes of two genera of wild Southern African omnivorous mammals. As predicted, we observed compositionally different diet and gut bacterial communities between the two host genera, which were strongly impacted by sampling localities. This supports that geography influences diet selection in wild viverrids with likely direct impacts on gut bacterial communities, aligning with previous findings from wild rodents (Wang et al., 2022) and primates (Zhao et al., 2018). We did not observe strong relationships between dietary item and gut bacterial ASV richness for either host genus, but we did detect strong associations between compositional similarity of consumed diets and the similarity in gut bacterial communities. These overall associations appear to be driven by correlations between specific diet items and microbial taxa that also differ by host genus. However, the latter should be interpreted with care, given the unknown nature of specific functions of these bacterial taxa and the uncertainty of whether diet‐associated taxa are transient microbes or symbionts (i.e., insect endosymbionts such as *Rickettsiella* and *Coxiella*). Moreover, given that the taxonomic proportions of diets were calculated based on presence/absence data, we might not be detecting more complex and fine‐scale associations between dietary taxa and microbes. Nevertheless, taken together, our results imply that composition/breadth of diets are strongly linked to gut bacterial communities in wild omnivorous mammals, aligning with previous findings from tropical birds (Bodawatta et al., 2022) and potentially revealing a major source of individual variation in gut microbiomes in wild mammals.

Mammals with similar diets tend to have similar gut microbiomes (Delsuc et al., 2014; Ley, Hamady, et al., 2008; Ley, Lozupone, et al., 2008; Muegge et al., 2011). However, although *C. civetta* and *Genetta* spp. are closely related hosts (Veron, 2007) that previously have been assigned to similar dietary niches, we found clear differences in both diet and gut bacterial community composition and diversity. This is consistent with work in other mammals where microbiome structure has often been attributed to the combination of host genetics and diet variation (Ley, Hamady, et al., 2008; Ley, Lozupone, et al., 2008; Mallott et al., 2018; Suzuki et al., 2019). Despite the detection of positive associations between diet and gut bacterial community similarities in both genera, we did not detect clear associations between dietary and microbial richness. This, along with the observed specific associations between diet orders and gut bacterial genera, suggests that the composition and variation in diets (i.e., what items are consumed) are more important in structuring viverrid gut microbiomes than diet richness. Supporting this, diet‐specific associations of gut microbes have also been found in wild primates (Mallott et al., 2018) and in diet manipulation work in both mammals (David et al., 2014) and birds (Bodawatta, Freiberga, et al., 2021). Coupled with this, observed influence of sampled locations on consumed diet and gut microbiome differences, implies that spatial variation in diet availability and consumption governs the gut microbial communities of these omnivorous mammals.

Metabarcoding provided novel insights into the dietary patterns of *C. Civetta* and *Genetta* spp., where we identified four times as many diet taxa as previous morphological studies have obtained (Amiard et al., 2015; Amroun et al., 2014; Bekele et al., 2008; Daniel et al., 2008; Sánchez et al., 2008). This implies that our current knowledge of dietary niches of these genera is likely underestimated. Although there were dietary overlaps between *C. civetta* and *Genetta* spp., their overall compositions were significantly different and dependent on location. *Genetta* spp. had overall more diverse diets and more plant taxa than previous morphological identifications have indicated (Breuer, 2008; Emmons, 1987). As *Genetta* spp. have previously been characterised as carnivores (Roberts et al., 2007; Rosalino & Santos‐Reis, 2002), this questions the applicability of using dietary niche classifications based on visual identification. Similarly, the diet of *C. civetta* was dominated by animal taxa, contradicting a previous study that suggested that it feeds mostly on grass and fruits (Mullu & Balakrishnan, 2015). However, identification of a diverse array of plant, vertebrate and invertebrate taxa in both genera imply that they belong to a broader omnivorous dietary guild (Admasu et al., 2004), underlining the importance of metabarcoding to decipher dietary niches of wild mammals.

Although developments in technologies have opened up new approaches in molecular biology for studying diets (Deiner et al., 2017; Pompanon et al., 2011), metabarcoding is not without flaws. It can provide information on the presence of an organism, but it does not reliably provide information on relative abundances (Alberdi et al., 2019; Neilsen et al., 2017). Therefore, we were only able to consider the frequency of a given diet taxon within or among samples. Furthermore, there is a risk of false positive detection of secondary diet items ‐ that is, organisms that were not consumed by the focal individual but by its prey, or components on prey items ingested for other reasons than nutrition (Bowser et al., 2013; Neilsen et al., 2017) or merely DNA contamination from the environment such as the air (Lynggaard et al., 2022). Like most ecological survey methods, metabarcoding also carries a risk of false negatives (Serrana et al., 2019), which can be introduced through primer bias, as two primers may provide vastly different taxonomic profiles. It is therefore important to choose primers with care, and the use of multiple primer sets can minimise these effects (Alberdi et al., 2017). Insufficient sampling depth was revealed in the species accumulation curves (Figure S1), indicating that the differences in sample sizes between the two host species could potentially have an effect on our conclusions. However, this may not have impacted our interpretations as we observed higher richness in the gut microbiomes and the consumed diets of *C. civetta*, despite smaller sample size.

## 5 CONCLUSIONS

Characterisation of animal diets has been revolutionised with metabarcoding techniques, providing deeper insights into variation and breadth of dietary niches that cannot be identified with traditional morphological methods. Our findings provide new insights into the more diverse and variable dietary consumption of omnivorous *C. civetta* and *Genetta* spp. than previously appreciated. Despite being sympatric, we detected genus‐specific dietary consumption, indicating non‐random and only partially overlapping dietary niches. This new knowledge on the feeding ecology of the focal genera is directly applicable to improve conservation strategies and preserve optimal habitats (and hence diet items) for these species (Chuang & Lee, 1997; Neilsen et al., 2017). Diet is one of the major drivers of mammalian gut microbial communities, but our results indicate that even closely related species under the same broad dietary guild classification would experience varying levels of associations between consumed diets and gut microbes. This implies that the effect of diet on mammalian microbiomes might be overestimated, when using broad dietary guilds. To improve our understanding of the magnitude of effects of diet on long‐ and short‐term gut bacterial associations with mammalian hosts, and diet contributions to microbiome variation, we need to understand realised dietary niches and consider geographic origins of individuals and species.

## AUTHOR CONTRIBUTIONS

**Malou B. Storm:** Conceptualization (equal); data curation (lead); formal analysis (equal); funding acquisition (equal); investigation (equal); methodology (equal); visualization (equal); writing – original draft (equal); writing – review and editing (equal). **Emilia M. R. Arfaoui:** Conceptualization (equal); data curation (equal); formal analysis (lead); funding acquisition (equal); investigation (equal); methodology (lead); validation (equal); visualization (equal); writing – original draft (equal); writing – review and editing (equal). **Phumlile Simelane:** Methodology (equal); resources (equal). **Jason Denlinger:** Methodology (equal); resources (equal). **Celine Alfredo Dias:** Methodology (equal); resources (equal). **Ana Gledis da Conceição:** Methodology (equal); resources (equal). **Ara Monadjem:** Investigation (equal); methodology (equal); resources (equal); supervision (equal); writing – review and editing (equal). **Kristine Bohmann:** Conceptualization (equal); formal analysis (equal); investigation (equal); methodology (equal); project administration (equal); resources (equal); supervision (equal); validation (equal); writing – original draft (equal); writing – review and editing (equal). **Michael Poulsen:** Conceptualization (equal); data curation (equal); funding acquisition (lead); methodology (equal); project administration (equal); resources (equal); supervision (equal); validation (equal); writing – original draft (equal); writing – review and editing (equal). **Kasun H. Bodawatta:** Conceptualization (equal); formal analysis (equal); methodology (equal); validation (equal); visualization (equal); writing – original draft (equal); writing – review and editing (equal).

## CONFLICT OF INTEREST STATEMENT

All the authors declare that there is no competing interest.

## Supporting information


Figure S1 and table captions.

Table S1‐S11.

Table S12.

## Data Availability

Sequence data is published in Erda with DOI https://doi.org/10.17894/ucph.db688f38‐95b7‐4b62‐902b‐86857a76c21e and R scripts and bioinformatic pipelines are available from https://github.com/emiliamrl/Viverridae_diet‐gutmicrobiome.
