# Supplementary material for: Diet components associated with specific bacterial taxa shape overall gut community compositions in omnivorous African viverrids
Source: Ecol Evol. 2024 Jul 11;14(7):e11486. doi: 10.1002/ece3.11486 (PMC11239323; doi:10.1002/ece3.11486)
Supplement: Supplementary file 1 — Figure S1 and table captions. [file ECE3-14-e11486-s001.docx]

**Supplemental Information for:**

**Diet components associated with specific bacterial taxa shape overall gut community compositions in omnivorous African viverrids**

Malou B. Storm^1,2#^, Emilia M. R. Arfaoui^1,2*#^, Phumlile Simelane^3^, Jason Denlinger^4^, Celine Alfredo Dias^4^, Ana Gledis da Conceição^4^, Ara Monadjem^3,5^, Kristine Bohmann^2^, Michael Poulsen^1^, and Kasun H. Bodawatta^2*^

^1^Section for Ecology and Evolution, Department of Biology, University of Copenhagen, Copenhagen, Denmark

^2^Section for Molecular Ecology and Evolution, Globe Institute, University of Copenhagen, Copenhagen, Denmark

^3^Department of Biological Sciences, University of Eswatini, Kwaluseni, Eswatini

^4^Gorongosa National Park, Mozambique

^5^Mammal Research Institute, Department of Zoology and Entomology, University of Pretoria, Hatfield, Pretoria, South Africa

*Correspondence: Emilia M. R. Arfaoui, email: emiliamrl93@gmail.com; Kasun H. Bodawatta, email: bodawata@sund.ku.dk

#Contributed equally

Supplementary figures


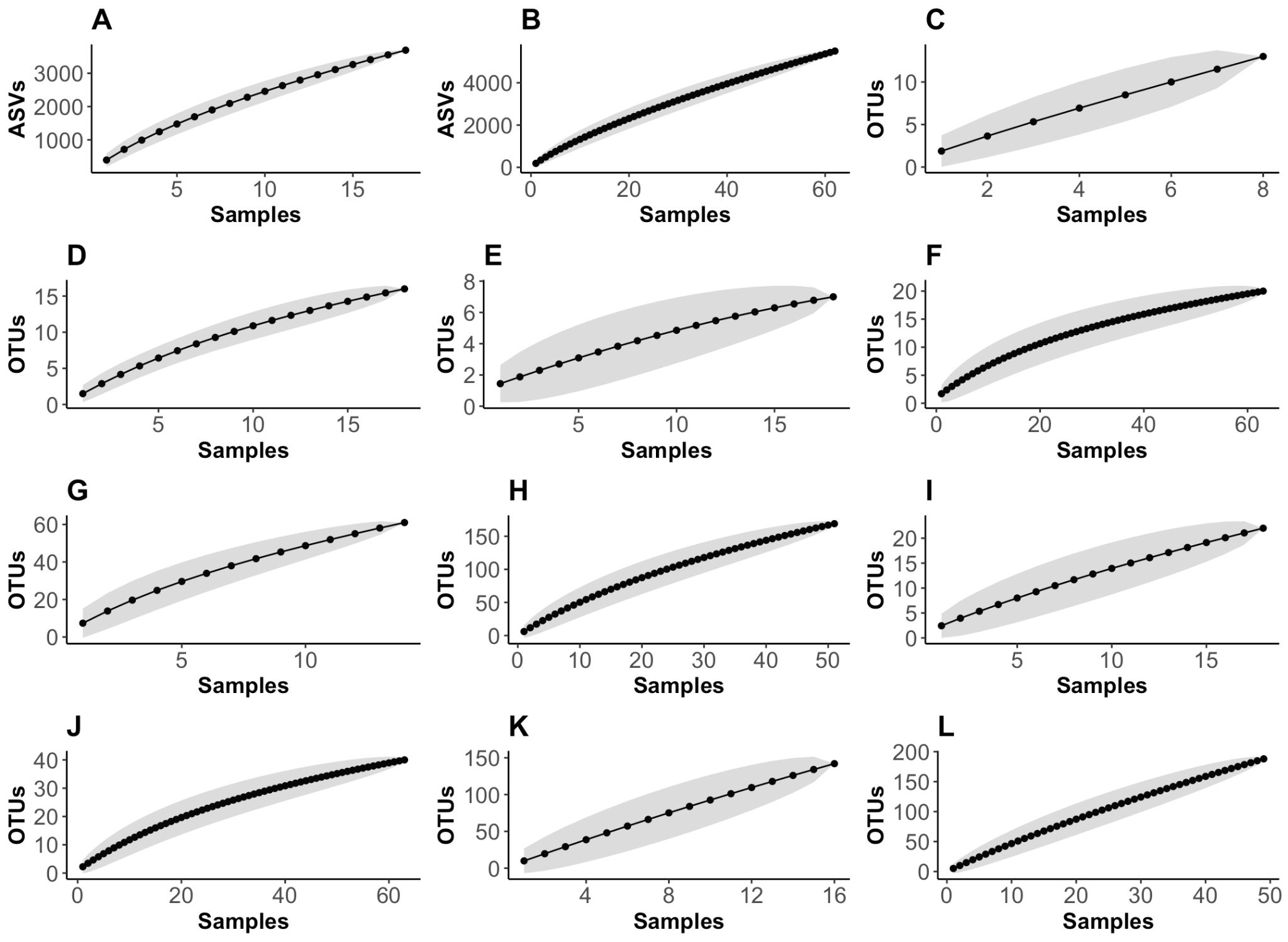


Figure S1. species accumulation curve of A) ASVs from bacterial 16S gene from *C. civetta*, B) ASVs from bacterial 16S gene from *Genetta* spp., C) OTUs from Coleop_16Sc and Coleop_16Sd marker from *C. civetta*, D) OTUs from Coleop_16Sc and Coleop_16Sd marker from *Genetta* spp., E) OTUs from 16S mamF and 16S mamR marker from *C. civetta*, F) OTUs from 16S mamF and 16S mamR marker from *Genetta* spp., G) OTUs from Trac01 and ITS 7A from *C. civetta*, H) OTUs from Trac01 and ITS 7A from *Genetta* spp., I) OTUs from RiazF and RiazR from *C. civetta*, J) OTUs from RiazF and RiazR from *Genetta* spp., K) OTUs from ZBJ-ArtF1c and ZBJ-ArtR2c from *C. civetta*, L) OTUs from ZBJ-ArtF1c and ZBJ-ArtR2c from *Genetta* spp. The grey areas show the standard deviation.

Supplementary Tables

**Table S1**. Complete list of *Civettictis civetta* and *Genetta* spp fecal samples collected at three Southern African locations (South Africa - SA, Mozambique - MZ, Eswatini - SW). Table also indicate the GPS coordinates of the latrines and whether the samples were sequenced for dietary and microbiome characterization

**Table S2**. Composition and recipe of RNAlater buffer used to preserve fecal samples in this study (Table is attached as a separate excel file).

**Table S3**. Museum tissue sample used to generate host species reference sequences and their museum id numbers from the Natural History Museum of Denmark (Table is attached as a separate excel file).

**Table S4**. Complete diet composition of *C. Civetta* and *Genetta* spp. on Class, Order, Family, Genus and Species level, including the sampling locations location (South Africa - SA, Mozambique - MZ, Eswatini - SW) (Table is attached as a separate excel file).

**Table S5.** OTU table of taxa identified with 16S mamF and 16S mamR primer pair, including their respective sequences (Table is attached as a separate excel file).

**Table S6.** OTU table of taxa identified with RiazF and RiazR primer pair, including their respective sequences (Table is attached as a separate excel file).

**Table S7.** OTU table of taxa identified with Coleop_16Sc and Coleop_16Sd primer pair, including their respective sequences (Table is attached as a separate excel file).

**Table S8.** OTU table of taxa identified with ZBJ-ArtF1c and ZBJ-ArtR2c primer pair, including their respective sequences (Table is attached as a separate excel file).

**Table S9.** OTU table of taxa identified with Trac01 and ITS 7A primer pair, including their respective sequences (Table is attached as a separate excel file).

**Table S10**. Bacterial ASV table with taxonomic assignments and their respective sequences (Table is attached as a separate excel file).

**Table S11**. Table indicating the overall distribution of bacterial sequences at bacterial phylum level and their proportion in *C. Civetta* and *Genetta* spp (Table is attached as a separate excel file).

**Table S12**. Pearson’s correlations between bacterial taxa at genus level and proportion of dietary items at order level in *C. Civetta* and *Genetta* spp. False discovery rate (FDR) adjusted significance of correlations are shown with asterisks (0.05 > * > 0.01, 0.01 > ** > 0.001, *** < 0.0001). **Tab 1** includes the correlations between bacteria and invertebrate orders in *C. Civetta*, while **Tab 2** include the correlations between bacteria and invertebrate orders in *Genetta* spp. **Tab 3** includes the correlations between bacteria and vertebrate orders in *C. Civetta*, while **Tab 4** include the correlations between bacteria and vertebrate orders in *Genetta* spp. **Tab 5** includes the correlations between bacteria and plant orders in *C. Civetta*, while **Tab 6** include the correlations between bacteria and plant orders in *Genetta* spp.
